# The Anti-Inflammatory Effect of Nigella sativa Toothpaste on *Porphyromonas gingivalis* Bacteria Through Decreased TNF-α, MMP-9, PGE-2 Expression in Wistar Rats

**DOI:** 10.1055/s-0043-1772700

**Published:** 2024-05-14

**Authors:** Ernie Maduratna, Desi Sandra Sari, Retno Puji Rahayu, Resgita Nadila Masya, Noor Adinar

**Affiliations:** 1 Department of Periodontics, Faculty of Dentistry Universitas Airlangga, Surabaya, Indonesia; 2 Department of Periodontics, Faculty of Dentistry, Universitas Jember, Jember, Indonesia; 3 Department of Anatomical Pathology, Faculty of Dentistry Universitas Airlangga, Surabaya, Indonesia; 4 Department of Restorative Dentistry, Faculty of Dentistry, Universiti Malaya, Kuala Lumpur, Malaysia

**Keywords:** anti-inflammatory, periodontitis, Nigella sativa

## Abstract

**Objective** The main principle in preventing periodontal disease is to improve oral hygiene. The bacteria that cause the onset of periodontal disease, one of which is the *Porphyromonas gingivalis* bacterium, causes inflammation. Persistent inflammation causes tissue damage and alveolar bone resorption by secreting proinflammatory cytokines, matrix metalloproteinase-9 (MMP-9), prostaglandin E2 (PGE-2), and anti-inflammatory cytokines. In this case, preventive treatment is needed, such as using toothpaste that contains anti-inflammatories so that the progression of the disease does not get worse. The traditional ingredient currently being developed is *Nigella sativa* , which has anti-inflammatory properties. Therefore, this study analyzes the potential of toothpaste containing *Nigella sativa* on the expression of tumor necrosis factor-α (TNF-α), MMP-9, and PGE-2 in the Wistar rat model induced by *Porphyromonas gingivalis* bacteria. This study aims to prove the potential of *Nigella sativa* toothpaste to decrease the expression of PGE-2, TNF-α, and MMP-9 in the gingiva of rats induced by *Porphyromonas gingivalis* bacteria.

**Materials and Methods** Forty-five healthy male Wistar rats were used, consisting of the negative control group, which was only injected with *Porphyromonas gingivalis* bacteria ATCC3322. The positive control group was given enzyme toothpaste, and the treatment group was assigned 1 mg of *Nigella sativa* paste using a microbrush for 30 seconds on the gingiva incisors mandibular with a circular motion, given two times a day for a week. Immunohistochemical to see the expression of TNF-α, PGE-2, and MMP-9. Parametric comparative analysis using a one-way analysis of variance test was performed to analyze differences between groups.

**Results and Discussion** *Nigella sativa* toothpaste significantly reduced proinflammatory cytokines, as seen through the expression of TNF-α, PGE-2, and MMP-9 on days 3, 5, and 7 ( *p* <0.05).

**Conclusion** In the limit of studied animal model, this trial indicates that giving toothpaste with black seed extract ( *Nigella sativa* ) could inhibit inflammatory mediators, as seen from the decreased expression of MMP-9, TNF-α, and PGE-2 seen from the 3rd, 5th, and 7th days.

## Introduction

Periodontal disease begins with an inflammatory condition of the gingiva called gingivitis due to the body's response to bacterial biofilms (also called plaque) adhering to tooth surfaces. One causative bacteria most often associated with periodontal disease is *Porphyromonas gingivalis* , a Gram-negative anaerobic bacterium. 1 2 Inflammation of the periodontal tissue causes redness of the gums because there is an increase in vascular proliferation and a decrease in keratinization due to compression pressure on the epithelium in the inflamed tissue. 3 The primary cause of periodontal inflammation is the presence of bacterial plaque on the teeth and gums. The bacteria in plaque produce a variety of toxins and enzymes that irritate the gums, causing them to become red, swollen, and bleed easily. 4 The immune system then responds to the bacterial invasion by releasing macrophage, neutrophils, and various inflammatory mediators, such as prostaglandins, cytokines, and chemokines, to combat the infection. 5 Tumor necrosis factor-alpha (TNF-α) and prostaglandin E2 (PGE2) are both mediators of inflammation that have been found to be elevated in periodontal disease. These cytokines are produced by immune cells called macrophages and are involved in the initiation and progression of the inflammatory response. 6 These cytokines can also stimulate the production of MMPs, which then degrade the extracellular matrix. MMP-9 has been shown to play a role in periodontal disease, a chronic inflammatory condition that affects the tissues surrounding and supporting the teeth. MMP-9 (matrix metalloproteinase 9) is an enzyme that plays a role in inflammation by breaking down extracellular matrix proteins such as collagen and elastin. MMP-9 is also involved in the activation of other inflammatory mediators, such as cytokines and chemokines. 7 Studies have found that individuals with periodontal disease have higher levels of MMP-9 in their gingival crevicular fluid (GCF) that is a fluid that fills the space between the gums and the tooth surfaces. Enzymes such as MMP-8 and MMP-9 play a significant role in the progression of periodontal disease by breaking down collagen in tissues and alveolar bone, leading to its degradation. 8

Treatment of periodontal disease aims to reduce the levels of aims to reduce the levels of microbial load. This can be achieved through mechanical therapies (scaling and root planning) and the use of antimicrobial agents. 9 10 Concomitant use of toothpaste or mouthwash with mechanical therapy can significantly improve therapeutic outcomes compared to scaling root planing (SRP) alone. 11 The current development uses traditional ingredients that are proven to be able to treat periodontal disease. Research by Mekhemar has shown that *Nigella sativa* has antibacterial and anti-inflammatory effects against bacteria that cause periodontitis. 12 These findings indicate that *Nigella sativa* could be used as ingredient in toothpaste for periodontal patients. In several previous studies, researchers tried to use *Nigella sativa* toothpaste as a preventive measure. *Nigella sativa* has a role as an antioxidant, anti-inflammatory, and ant ischemic, and it has antibacterial activity. 13 The thymoquinone content has been experimentally proven to show an anti-inflammatory function, which can reduce nitric oxide levels and suppress proinflammatory cytokines. 12 With all the benefits of traditional medicines such as *Nigella sativa* as toothpaste, it is hoped that this can reduce inflammation by becoming an additional physical barrier for the epithelium in protecting the oral tissues from bacterial invasion. 13 A study by Setiawatie et al stated that giving *Nigella sativa* extract to toothpaste inhibited the activity of inflammatory mediators. 14 Based on this background, an effective method is needed to prevent and treat periodontal disease. This study was conducted to prove the potential of *Nigella sativa* toothpaste as an anti-inflammatory in the gingiva of rats induced by *Porphyromonas gingivalis* bacteria by decreasing the expression of PGE-2, TNF-α, MMP-9.

## Materials and Methods

Forty-five male Wistar rats ( *Rattus norvegicus* ) aged 5 to 6 months (bodyweight, 250–350 g) were adapted to the laboratory environment for at least 1 week and housed under standard laboratory conditions. All procedures conducted on animals were approved by the Health Research Ethical Clearance Commission at the Faculty of Dental Medicine, Universitas Airlangga (approval number: 733/HRECC.FODM/IX/2022). Briefly, *Porphyromonas gingivalis* (Pg ATCC 33277 PK/5, Thermo Scientific; 1 × 10 colony-forming units (CFU) in 20 μL of phosphate buffered saline) was injected locally on the gingiva of the rats under the mesial right and left mandibular incisor gingival sulcus using a 0.5-mL syringe. The injection was performed on first day for 7 days. The initial signs of gingivitis, which included a reddish pigmentation of the gingiva, swelling of the interdental incisive central mandibular area, were observed

The experimental rats in the control and treatment groups were randomly divided into nine groups. Before inducing *Porphyromonas gingivalis* , anesthesia was performed using a combination of ketamine and xylazine. The dose given is ketamine 0 to 75 mg/kg and xylazine 5 to 10 mg/kg in a 1: 1 ratio intramuscularly posterior to the right with a duration of anesthetic effect of about 20 to 30 minutes. *Porphyromonas gingivalis* ATCC 33 277 bacteria were injected locally into the gingiva of rats as much as 0.03 mL with a concentration of 2 × 106 CFU/mL under the gingival sulcus of the mandibular incisors on the right and left mesial sections done once in the morning for 1 week with a 30 G needle (BD).

*Nigella sativa* toothpaste is a toothpaste made based on an elemental toothpaste composition added to *Nigella sativa* extract with a composition of *Calcium Carbonate, Sorbitol, Aqua, Carboxymethyl Cellulose, Silica, Titanium Dioxide, Nigella sativa Seed Oil 3%, Sodium Benzoate, Sodium Lauroyl Sarcosinate, Flavor, Flavor, Flavor, Allantoin, Sodium Saccharin, Methylparaben,* and *CI 42090* . One milligram of *Nigella sativa* toothpaste was administered twice a day simultaneously with the induction of *Porphyromonas gingivalis* in the gingival sulcus of the mandibular incisors.

This study that aims to prove the expression of PGE-2, TNF-α, and MMP-9 used animals that were randomly divided into three groups as follows: control group rats that are PG only group were sacrificed after day 3, 5, and 7; PG + toothpaste comprising enzyme were sacrificed after day 3, 5, and 7; PG + *Nigella sativa* toothpaste which comprised rats that received *Nigella sativa* toothpaste were sacrificed after day 3, 5, and 7.

In this study, statistical analysis was performed using GraphPad Prism software. The normality of the data was tested using the Shapiro–Wilk test, and the homogeneity was checked with Levene's test. If the data meets the requirements for a parametric test, a one-way analysis of variance will be performed to detect differences between groups, with a significance level of *p* -value less than 0.05. If the data does not meet the criteria for a parametric test, a nonparametric Kruskal–Wallis test will be conducted instead, also with a significance level of *p* -value less than 0.05. To identify specific differences between groups, the multiple comparison post-hoc tests with Tukey's honestly significant difference (HSD) will be conducted. The results of the normality and homogeneity tests found that the distribution of the research data met the requirements, namely normal data distribution ( *p* > 0.05) and homogeneous data with a value ( *p* > 0.05), so the parametric comparative test was continued.

## Result

The results of an examination of macrophage cells expressing TNF-α on days 3, 5 7 using immunohistochemical techniques can be seen in Table 1 . The analysis results in Table 1 show the highest average expression of TNF-α on day 5 and 7 in the control group.

**Table 1:** The mean and SD values of TNF-α expression on day 3, 5, and 7 of macrophages in the control (K) and treatment (P) groups

TNF-α (mg/mL)			
Group	3 days	5 days	7 days
	Mean ± SD	Mean ± SD	Mean ± SD
Control group	9.80 ± 1.720	12 ± 2.756	12 ± 1.788
Enzyme toothpaste group	4 ± 1.414	4 ± 1.414	3.2 ± 1.661
Nigella sativa toothpaste group	5.6 ± 1.019	4 ± 1.414	4 ± 1.661

Abbreviations: SD, standard deviation; TNF-α, tumor necrosis factor-alpha.

Table 2 displays the results of examining macrophage cells that express PGE-2 on days 3, 5, and 7. The analysis reveals that the control group had the highest average expression of PGE-2 on day 7.

**Table 2:** The mean and SD values of PGE-2 expression on day 3, 5, and 7 of macrophages in the control (K) and treatment (P) groups

PGE-2 (mg/mL)			
Group	3 days	5 days	7 days
	Mean ± SD	Mean ± SD	Mean ± SD
Control group	9.20 ± 0.9797	11 ± 1.141	13.6 ± 2.059
Enzyme toothpaste group	3.4 ± 1.496	3.4 ± 1.019	3 ± 0.632
Nigella sativa toothpaste group	5.8 ± 10.7483	5.60 ± 1.356	3.4 ± 1.864

Abbreviations: PGE-2, prostaglandin E2; SD, standard deviation.

Table 3 presents the findings from the investigation of PGE-2 expression in macrophage cells on days 3, 5, and 7. The data indicates that the control group had the highest average level of MMP-9 expression on day 7.

**Table 3:** The mean and SD values of MMP-9 expression on day 3,5, and 7 of macrophages in the control (K) and treatment (P) groups

MMP-9 (mg/mL)			
Group	3 days	5 days	7 days
	Mean ± SD	Mean ± SD	Mean ± SD
Control group	9.4 ± 1.356	12.6 ± 2.154	12.8 ± 1.720
Enzyme toothpaste group	4.4 ± 1.019	4 ± 1.414	2.4 ± 1.019
Nigella sativa toothpaste group	5.8 ± 0.748	5.6 ± 1.356	3.4 ± 1.854

Abbreviations: MMP-9, matrix metalloproteinase-9; SD, standard deviation.

## TNF-α Expression

**Fig. 1:**
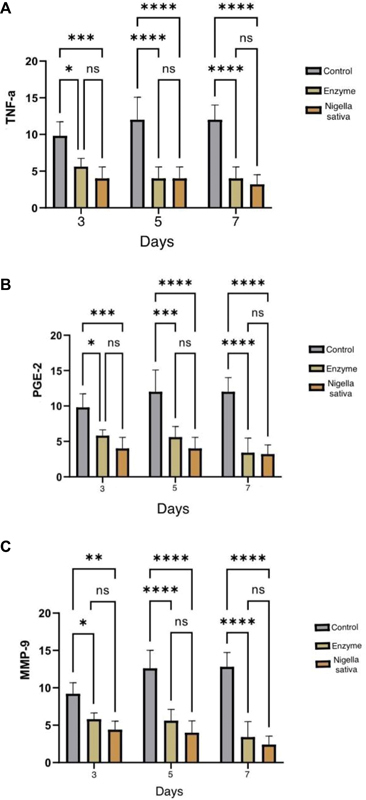
( **A–C** ) The mean value of tumor necrosis factor-alpha (TNF-α), prostaglandin E2 (PGE-2), and matrix metalloproteinase-9 (MMP-9) on days 3, 5, and 7 of macrophages in the control (K) and treatment (P) groups (****: *p* < 0.0000; ***: *p* < 0.001); **: *p* < 0.01; *: *p* < 0.05; ns: insignificant difference).

Fig. 1A shows the results of the TNF-α expression using Tukey's HSD post-hoc test. There was a significant difference in Fig. 1A between the negative control and *Nigella sativa* on the 3rd, 5th, and 7th day, respectively ( *p* < 0.05; *p* < 0.000; *p* < 0.0000). Furthermore, significant differences also occurred in the negative control variables with enzymes on the 3rd, 5th, and 7th day, respectively ( *p* <0.001; *p* <0.000; *p* <0.000) and finally, there was no significant difference between the enzyme and *Nigella sativa* ( *p* > 0.05).

## PGE-2 Expression

The results of the PGE-2 expression study using Tukey's HSD post-hoc test are depicted in Fig. 1B . There was a marked difference between the negative control and *Nigella sativa* on the 3rd, 5th, and 7th day as evidenced by *p* -values of less than 0.05, less than 0.001, and less than 0.000, respectively. Additionally, significant variations were also identified between the negative control and enzyme variables on the 3rd, 5th, and 7th day, as indicated by *p* -values of less than 0.001, less than 0.000, and less than 0.000, respectively. Finally, no significant discrepancy was detected between the enzyme and *Nigella sativa* , with a *p* -value of more than 0.05.

## MMP-9 Expression

The findings of the MMP-9 expression analysis using Tukey's HSD post-hoc test are presented in Fig. 1C . The results revealed that there was a notable discrepancy in the expression levels between the negative control and *Nigella sativa* on the 3rd, 5th, and 7th day. This difference was statistically significant with *p* -values of less than 0.05, less than 0.000, and less than 0.000, respectively. Furthermore, the study also showed significant differences between the negative control and enzyme variables on the same days, with *p* -values of less than 0.01, less than 0.000, and less than 0.000, respectively. However, it was found that there was no significant difference in expression levels between the enzyme and *Nigella sativa* , as indicated by a *p* -value of more than 0.05.

<insert figure 1 here>

**Fig. 2:**
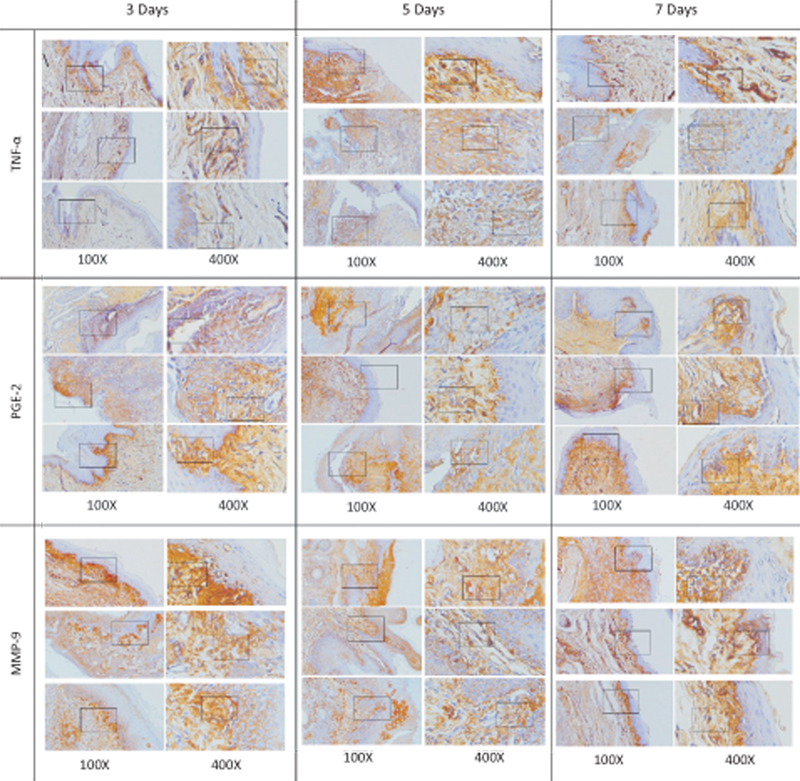
The results of immunohistochemical examination showed tumor necrosis factor-alpha (TNF-α), prostaglandin E2 (PGE-2), and matrix metalloproteinase-9 (MMP-9) expression on days 3, 5, and 7 from macrophages indicated by brown cells (black arrows) in each group (magnification 100x, 400x).

The histopathology view of the TNF-α, PGE-2, and MMP-9 expression was observed using an inverted light microscope with 100x, 400x as can be seen in Fig. 2 .

## Discussion

This study found that the expression of TNF-α, PGE-2, and MMP-9 in the treatment group decreased compared to the control group and was shown by the decrease in the number of cells in the treatment group, significantly different from the control group on 3 days of observation ( *p* <0.0001). *Nigella sativa* belongs to the family Ranunculaceae, a historical plant known for traditional medicines used as a therapy for several diseases. *Nigella sativa* also known as black cumin or black seed is a small flowering plant that is native to the Mediterranean and Southwest Asia. The seeds of the plant have been traditionally used in Middle Eastern and Asian cuisine and have a long history of use in traditional medicine. Research has shown that the active compounds in *Nigella sativa* , such as thymoquinone, have anti-inflammatory effects by inhibiting the production of inflammatory mediators, such as cytokines and prostaglandins. 12

Some studies have also shown that *Nigella sativa* can inhibit the growth of certain bacteria that are associated with periodontal disease. It has also been observed to reduce the level of inflammatory markers such as C-reactive protein, TNF-α, and interleukin-6 (IL-6), which are indicative of periodontal inflammation. 14 15 Research by Setiawatie et al showed that *Nigella sativa* toothpaste treatment could reduce the number of neutrophils and macrophage cells that act as phagocytic cells which help improve tissue healing. 14 The decrease in the number of inflammatory mediator cells in this study was seen in the individual results of the expression of TNF-α, PGE-2, and MMP-9 on days 3, 5, and 7 that showed significant results ( *p* <0.05), which proved that the administration of the paste *Nigella sativa* teeth could prevent inflammation. Thymoquinone is the active component that ranges from 30 to 48% in *Nigella sativa* seeds. 16 The active ingredient thymoquinone can reduce proinflammatory mediators such as IL-1β, IL-6, TNF-α, interferon-gamma, and PGE-2 by mediating COX-2 expression so that it functions as an anti-inflammatory through Mitogen-activated protein kinase (MAPK) and nuclear factor kappa B (NF-κB) signaling. Thymoquinone suppresses NF-κB activation induced by various carcinogens and inflammatory agents, and NF-κB inhibition is due to the inhibition of IkB kinase (IKK) activation, leading to suppression of NF kappa B inhibitor alpha (IκBα) phosphorylation and degradation. 17 The intense antibacterial activity of *Nigella sativa* can be used as a therapeutic agent or adjuvant in bacterial infections.

This study also used a positive control, namely toothpaste containing enzymes that also decreased the expression of MMP-9, TNF-α, and PGE-2, which was significant to the negative control but not significantly different from toothpaste with *Nigella sativa* . Enzyme toothpaste contained lactoperoxidase, amyloglucosidase, and glucose oxidase enzymes in this study. Lactoperoxidase, an enzyme secreted by saliva, has an antimicrobial effect against bacteria. Amyloglucosidase and glucose oxidase work together to produce hydrogen peroxide. Lactoperoxidase exhibits an antimicrobial effect by catalyzing the conversion of thiocyanate to hypothiocyanite. 18 The formation of hypothiocyanite has many beneficial factors for the host because it targets a variety of microorganisms and also acts as an anti-inflammatory by accumulating hypochlorous acid produced by neutrophils, a significant cause of neutrophil-mediated oxidative tissue damage. 19 According to research by Welk et al, lactoperoxidase is an antioxidant that can reduce the amount of reactive oxygen species during the inflammatory process so that it can protect the structure of the periodontal tissue. 20 In addition, the lysozyme enzyme contained in enzyme toothpaste can attack bacteria by attacking their cell walls so that they become porous, causing the bacteria to lose cell fluids and eventually become dead cells. 21 This enzyme functions effectively as an antibacterial when it works with lactoferrin and sIgA. The lactoferrin enzyme in the enzyme paste also acts as an antibacterial and tends to be bacteriostatic by binding to Fe3+ ions, which are needed for the growth of microorganisms. 22 Enzyme toothpaste can prevent infection so that it can indirectly reduce inflammatory activity, as illustrated by the results of this study, where there was no significant difference between the expression of PGE-2, MMP-9, and TNF-α in the treatment group and the enzyme group. Therefore, it can be concluded that toothpaste with *Nigella sativa* extract and enzyme content has an anti-inflammatory function. Toothpaste containing the enzyme in this study was a positive control, also having a significant difference in the expression of TNF-α, MMP-9, and PGE-2. This study also proves that there is no significant difference between enzyme toothpaste and toothpaste with *Nigella sativa* extract, so they have the same function to help reduce inflammatory activity.

Limitation of this study is use only male Wistar rats. We use male rats, because male rats do not experience hormonal changes that will affect the periodontal condition. This study has a potential benefit for periodontal disease patients to inhibit inflammatory mediators.

## Conclusion

The effects of using toothpaste with *Nigella sativa extract* on *Porphyromonas gingivalis* bacteria injected in Wistar Rats were examined in this study. The *Nigella sativa* extract toothpaste inhibits anti-inflammatory mediators (TNF-α, MMP-9, and PGE-2).
